# PlanHab: Hypoxia counteracts the erythropoietin suppression, but seems to exaggerate the plasma volume reduction induced by 3 weeks of bed rest

**DOI:** 10.14814/phy2.12760

**Published:** 2016-04-13

**Authors:** Michail E. Keramidas, Igor B. Mekjavic, Roger Kölegård, Alexander Choukèr, Claudia Strewe, Ola Eiken

**Affiliations:** ^1^ Department of Environmental PhysiologySwedish Aerospace Physiology CenterSchool of Technology and HealthRoyal Institute of TechnologyStockholmSweden; ^2^ Department of AutomationBiocybernetics and RoboticsJozef Stefan InstituteLjubljanaSlovenia; ^3^ Department of AnaesthesiologyStress and Immunology LabUniversity of MunichMunichGermany

**Keywords:** Blood volume, confinement, hypovolemia, microgravity, renin

## Abstract

The study examined the distinct and synergistic effects of hypoxia and bed rest on the erythropoietin (EPO) concentration and relative changes in plasma volume (PV). Eleven healthy male lowlanders underwent three 21‐day confinement periods, in a counterbalanced order: (1) normoxic bed rest (NBR; P_I_O_2_: 133.1 ± 0.3 mmHg); (2) hypoxic bed rest (HBR; P_I_O_2_: 90.0 ± 0.4 mmHg, ambient simulated altitude of ~4000 m); and (3) hypoxic ambulation (HAMB; P_I_O_2_: 90.0 ± 0.4 mmHg). Blood samples were collected before, during (days 2, 5, 14, and 21) and 2 days after each confinement to determine EPO concentration. Qualitative differences in PV changes were also estimated by changes in hematocrit and hemoglobin concentration along with concomitant changes in plasma renin concentration. NBR caused an initial reduction in EPO by ~39% (*P *=* *0.04). By contrast, HBR enhanced EPO (*P *=* *0.001), but the increase was less than that induced by HAMB (*P *<* *0.01). All three confinements caused a significant reduction in PV (*P *<* *0.05), with a substantially greater drop in HBR than in the other conditions (*P *<* *0.001). Thus, present results suggest that hypoxia prevents the EPO suppression, whereas it seems to exaggerate the PV reduction induced by bed rest.

## Introduction

Erythropoietin (EPO), a glycoprotein hormone produced by the adult kidney (Bauer and Kurtz 1989; Lundby et al. 2014) whose main function is to regulate the production rate of red blood cell volume, is governed primarily by the relative amount of O_2_ available to the tissues (Jelkmann 2011). It is well established that exposure to hypoxia prompts a rather rapid increase in EPO concentration (Eckardt et al. 1989; Knaupp et al. 1992), which reaches its zenith after 3–4 days, and is thereafter followed by a gradual decline toward the pre‐hypoxia levels (Gunga et al. 1994; Berglund et al. 2002). There is also evidence to suggest that the EPO secretion is modulated by changes in blood volume (Berglund et al. 1987; Ehmke et al. 1995; Breymann et al. 2000); albeit the underlying mechanism is not yet clear (cf. Kirsch et al. 2005). Thus, a prolonged period of bed rest suppresses EPO synthesis, presumably due to the central venous pressure increase resulting from the thoraco‐cephalad blood volume shift at the initial stage of recumbency (Gunga et al. 1996). Yet, whether, and to what extent, a hypoxic stimulus superimposed on bed rest would counteract the bed rest‐induced erythropoietic suppression remains unclear.

Exposure to high altitude causes a rapid contraction in plasma volume (PV) (Pugh 1964; Singh et al. 1990; Sawka et al. 1996; Siebenmann et al. 2015); a response that has been attributed to several mechanisms, viz. dehydration, diuresis, plasma protein loss, and inhibition of the renin–angiotensin–aldosterone axis (cf. Hoyt and Honig 1996). A prolonged period of bed rest also leads to a substantial reduction in PV (Greenleaf 1984; Johansen et al. 1997), which is mainly attributable to the increased secretion of atrial natriuretic peptide (ANP), and in part, to the reduction of total circulating plasma proteins (Fortney et al. 1996; Convertino 2007). Loeppky et al. (1993) have suggested that the coexistence of the hypoxia and bed rest stressors might have an additive effect on the PV response, thereby leading to a greater degree of hypovolemia than that induced by either hypoxia or bed rest per se; however, this still remains unsettled.

Accordingly, the purpose of the study was to examine the effects of 21 days of hypoxia and horizontal bed rest, alone and in combination, on EPO concentration, and on qualitative interconfinement differences in PV changes. We hypothesized that normobaric hypoxia would: (1) counteract the bed rest‐induced suppression in EPO concentration; and (2) augment the bed rest‐induced reduction in PV.

## Materials and Methods

The study was part of the “PlanHab: Planetary Habitat Simulation” project investigating the effects of a 21‐day hypoxic bed rest on several functions of cardiorespiratory, musculoskeletal, thermoregulatory, and neurohumoral systems. Complete details of the study have been reported previously, including the selection procedures and characteristics of the subjects, and a thorough description of the three confinements (see Debevec et al. 2014; Ciuha et al. 2015).

### Subjects

Eleven healthy males (age: 27 ± 6 years, stature: 179.9 ± 3.2 cm, body mass: 76.6 ± 11.9 kg, body fat: 21 ± 5%, peak O_2_ uptake: 3.1 ± 0.6 L min^−1^) participated in the study. Prior to the onset of the study, subjects had a thorough physical examination, and their participation was subject to a physician's approval. None of them had history of any cardiorespiratory, hematological, or renal disease. They were physically active on a recreational basis, and had not been exposed to altitudes >1000 m during the 2 months preceding the experiments. Subjects were informed in detail about the experimental procedures before giving their written consent to participate, and were aware that they could terminate their participation in the study at any time. The experimental protocol was approved by the National Committee for Medical Ethics at the Ministry of Health of the Republic of Slovenia and conformed to the Declaration of Helsinki.

### Experimental protocol

The study was conducted at the Olympic Sports Center Planica (Rateče, Slovenia) that is situated at an altitude of 940 m. To determine the distinct effects of hypoxia and bed rest, and their synergy, all subjects underwent three 21‐day confinement periods, in a counterbalanced order and separated by a washout period of at least 4 months: (1) a normoxic bed rest (NBR), during which they were breathing room‐air [fraction of O_2_ (FO_2_): 0.21, partial pressure of inspired O_2_ (PIO2): 133.1 ± 0.3 mmHg]; (2) a hypoxic bed rest (HBR), while they were continuously inspiring a hypoxic gas mixture (FO_2_: 0.14, P_I_O_2_: 90.0 ± 0.4 mmHg, ambient simulated altitude of ~4000 m); and (3) a hypoxic ambulation (HAMB), during which subjects were exposed to the same hypoxic environment as in the HBR. Throughout the three confinements, subjects were under 24‐h care, and their daily well‐being was monitored by the medical staff. Subjects consumed a standardized diet, and were allowed to drink water and tea ad libitum (see Debevec et al. 2014).

Venous blood was drawn from an antecubital vein on six occasions: before (Pre), during (on days 2, 5, 14 and 21), and 2 days after (R2) each confinement period. The blood was always collected at 0730 h in the morning, while subjects rested in a horizontal, supine position after an overnight fast. The samples were analyzed for serum erythropoietin (EPO) concentration, reticulocyte count, hemoglobin concentration ([Hb]), hematocrit (Hct), total red blood cells (RBCs), and plasma renin concentration (see below for details). The blood samples for EPO and renin determination were centrifuged, and the plasma and serum were frozen to −80°C for subsequent analyses. All hematological variables were determined in duplicate by researchers, who were blinded as regards the intervention.

Following the blood sampling, systolic (SAP) and diastolic (DAP) arterial pressure was measured with a noninvasive oscillometric automated sphygmomanometer (Omron M6, Kyoto, Japan). The mean arterial pressure (MAP) was calculated accordingly. Heart rate (HR) and capillary oxyhemoglobin saturation (SpO_2_) were recorded with short‐range telemetry (iBody, Wahoo Fitness, Atlanta, USA) and a finger pulse oxymeter (3100 WristOx, Nonin Medicals, Minessota), respectively.

#### Bed rest and ambulatory confinements

During NBR and HBR, subjects were confined to a strict horizontal position, and were not allowed to perform any exercise or strenuous muscle contractions. They were allowed to move their arms in and above the horizontal plane and to lean on their elbows during eating, personal hygiene, and transfer between bed and gurney. During HAMB, subjects were allowed to move freely within the hypoxic living area and were encouraged to be active, so as to maintain their normal daily activity level. To that purpose, they also performed two 30‐min bouts of light exercise (average HR: 124 ± 9 beats min^−1^), such as stepping, cycling, or dancing, once in the morning and once in the afternoon.

During HBR and HAMB, hypoxia in the confinement area was achieved using an O_2_ dilution system (b‐Cat, Tiel, the Netherlands) based on the vacuum pressure swing adsorption principle. The O_2_ level was monitored continuously with O_2_ sensors (PGM‐1100; Rae Systems, San Jose, California).

#### Hematological analyses

EPO concentration was determined by sandwich enzyme‐linked immunoassay (Quantikine IVD EPO ELISA; R&D Systems, Minneapolis, MN) using 100 *μ*L of serum. Optical density was quantified on a SPECTRA_max_TM PLUS^384^ microplate spectrophotometer (Molecular Devices Corporation, 1311 Orleans Drive, Sunnyvale, California) set at 450 nm and corrected at 600 nm. The estimated coefficient of variation of the analysis was 2.2%.

[Hb], Hct, RBCs, and reticulocyte counts were analyzed with an automated laser‐based hematology analyser (Advia 120; Siemens, Munich, Germany) within 8 h after the blood sampling using clinical laboratory standards.

Plasma renin concentration was quantified by an automated sandwich chemoluminescence immunoassay (LIAISON XL, DiaSorin S.p.A., Saluggia, Italy). The estimated coefficient of variation of the analysis was 4.5%.

The interconfinement differences in PV changes were estimated qualitatively from changes in Hct and [Hb] (Dill and Costill 1974; Johansen et al. 1997). To ensure that the interconfinement grading of PV changes thus obtained were not detrimentally confounded by neoerythrocytosis, results were compared with the concomitant changes in plasma renin concentration (Gauer and Henry 1976). Thus, any exaggerated PV drop was considered factual, only if accompanied by an exaggerated increase in renin concentration.

### Statistical analysis

Statistical analyses were performed using Statistica 8.0 (StatSoft, Tulsa, OK). All data are reported as mean ± SD. Analysis of the normal distribution of the data was performed with the Kolmogorov–Smirnov test, and the homoscedasticity was tested using the Levene's test. Thereafter, a two‐way (confinement × time) general linear model repeated measures ANOVA was used to examine the differences in all hematological variables. Mauchly's test was conducted to assess for sphericity, and the Greenhouse‐Geisser *ε* correction was used to adjust the degrees of freedom, when the assumption of sphericity was not satisfied. When ANOVA revealed significant *F*‐ratio for interaction, pairwise comparisons were performed with Tukey HSD *post hoc* test to assess differences between confinements. Changes from the prevalue within each confinement were analyzed with a Dunnett test. Partial eta‐squared (*η* ^2^_p_) effect sizes were determined for all significant interactions and main effects revealed by the repeated measures ANOVAs (values for *η* ^2^_p_ of ≤0.02, ≤0.13, and ≥0.26 are considered as small, moderate, and large, respectively). Cohen's *d* effect sizes were also computed [*d *=* (M*_*i*_ *− M*_*j*_ *)/*SD_pooled_] for select contrasts (values for *d* of ≤0.2, ≤0.5, and ≥0.8 are considered as small, moderate, and large, respectively) (Cohen 1988). A moderate to large effect size represents a functional effect of the confinement. Statistical power calculations in this study ranged from 0.71 to 1.00. The alpha level of significance was set a priori at 0.05.

## Results

The mean values of HR, SAP, DAP, MAP, and SpO_2_ during each confinement are presented in Table 1. The daily values of HR and SpO_2_ have been discussed in detail previously (see Debevec et al. 2014). NBR and HAMB did not alter HR. However, HBR increased HR (*P *=* *0.02); HR was higher in HBR than NBR (*P *<* *0.01). None of the confinements changed SAP, but all confinements increased DAP (*P *<* *0.01). DAP tended to be higher in the HBR than the NBR, albeit the difference was not statistically significant (*P *=* *0.12). MAP was increased by HBR (*P *=* *0.01), and there was a statistical tendency for an elevated MAP in NBR (*P *=* *0.06). HBR and HAMB reduced SpO_2_ (*P *<* *0.001), which was lower in these conditions than in NBR (*P *<* *0.001).

**Table 1:** Systolic (SAP), diastolic (DAP) and mean (MAP) arterial pressure, heart rate (HR), and capillary oxyhemoglobin saturation (SpO_2_) before, during, and after the 21‐day hypoxic bed rest (HBR), normoxic bed rest (NBR), and hypoxic ambulation (HAMB)

	Pre	HBR	R2	Pre	NBR	R2	Pre	HAMB	R2
HR (beats min^−1^)	64 ± 12	73 ± 7†, *	68 ± 9	64 ± 11	61 ± 8	66 ± 8	64 ± 9	69 ± 7	66 ± 16
SAP (mmHg)	115 ± 11	120 ± 9	111 ± 14	112 ± 7	117 ± 6	114 ± 8	113 ± 13	116 ± 8	110 ± 11
DAP (mmHg)	66 ± 5	75 ± 7†	68 ± 12	62 ± 6	69 ± 6†	66 ± 5	64 ± 5	71 ± 6†	67 ± 9
MAP (mmHg)	82 ± 7	90 ± 7†	83 ± 13	78 ± 5	85 ± 5	82 ± 6	81 ± 7	86 ± 6	82 ± 8
SpO_2_ (%)	97 ± 1	88 ± 1†, *	97 ± 1	98 ± 0	97 ± 1#	98 ± 1	97 ± 1	87 ± 1†	97 ± 1

Values are ± SD.

^†^Significantly different from the Pre. *Significant differences between HBR and NBR. ^#^Significant differences between NBR and HAMB. (*P *<* *0.05).

**Table 2:** Count of red blood cells per unit volume of blood (RBCs) before (Pre), during (day 2, 5, 14 and 21), and after (R2) the 21‐day hypoxic bed rest (HBR), normoxic bed rest (NBR), and hypoxic ambulation (HAMB)

	Pre	Day 2	Day 5	Day 14	Day 21	R2
RBCs (×10^12^ L^−1^)						
HBR	5.16 ± 0.29	5.58 ± 0.33†	5.90 ± 0.39†, §	6.22 ± 0.36†, *, §	6.08 ± 0.49†, *, §	5.34 ± 0.49
NBR	5.10 ± 0.38	5.53 ± 0.40†	5.55 ± 0.40†	5.59 ± 0.37†	5.48 ± 0.47†	5.39 ± 0.88†
HAMB	5.01 ± 0.33	5.41 ± 0.28†	5.41 ± 0.47†	5.54 ± 0.39†	5.64 ± 0.47†	5.10 ± 0.41

Values are ± SD.

^†^Significantly different from the Pre. *Significant differences between HBR and NBR. ^§^Significant differences between HBR and HAMB. (*P *<* *0.001).

### Serum erythropoietin

**Figure 1:**
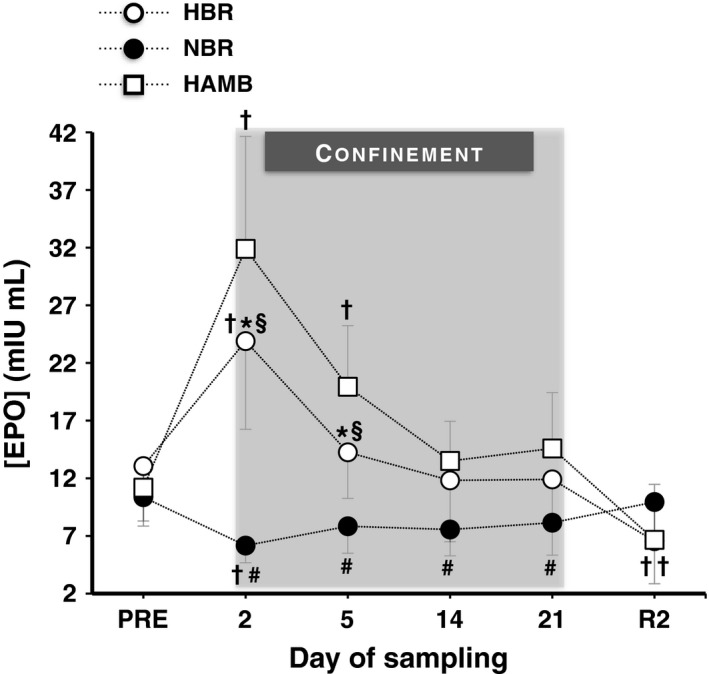
Absolute values of serum erythropoietin concentration ([EPO]) before (Pre), during (day 2, 5, 14 and 21), and after (R2) the 21‐day hypoxic bed rest (HBR), normoxic bed rest (NBR), and hypoxic ambulation (HAMB). Values are mean ± SD. ^†^Significantly different from the Pre. *Significant differences between HBR and NBR. ^§^Significant differences between HBR and HAMB. ^#^Significant differences between NBR and HAMB. (*P *<* *0.001).

**Figure 2:**
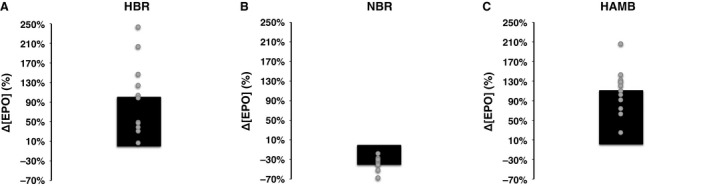
Mean and individual changes from prevalues of serum erythropoietin concentration ([EPO]) on day 2 of the 21‐day hypoxic bed rest (HBR; A), normoxic bed rest (NBR; B), and hypoxic ambulation (HAMB; C). Values are mean ± SD.

The mean absolute values of EPO concentration during the three confinements are presented in Figure 1. Figure 2 illustrates the mean and individual relative changes of EPO on day 2 of each confinement. There was a confinement × time interaction (*P *<* *0.001, *η* ^2^_p_ **=* *0.73), and a main effect for confinement (*P *<* *0.001, *η* ^2^_p_ **=* *0.83) and time (*P *<* *0.001, *η* ^2^_p_ **=* *0.79). HAMB increased EPO on day 2 and 5 (*P *<* *0.001). HBR enhanced EPO on day 2 (*P *<* *0.001, *d *=* *1.66); the increase was less in HBR than in HAMB (*P *≤* *0.01). Contrary to the hypoxic confinements, NBR caused an initial reduction in EPO by ~39% (*P *=* *0.04, *d *=* *2.33). EPO levels were significantly lower in NBR compared to in HBR on days 2 and 5 (*P *≤* *0.01), and to in HAMB throughout the confinement (*P *≤* *0.001). After the cessation of both hypoxic confinements, EPO was decreased by ~48% (*P *<* *0.05); no such drop was observed following NBR.

### Reticulocytes

**Figure 3:**
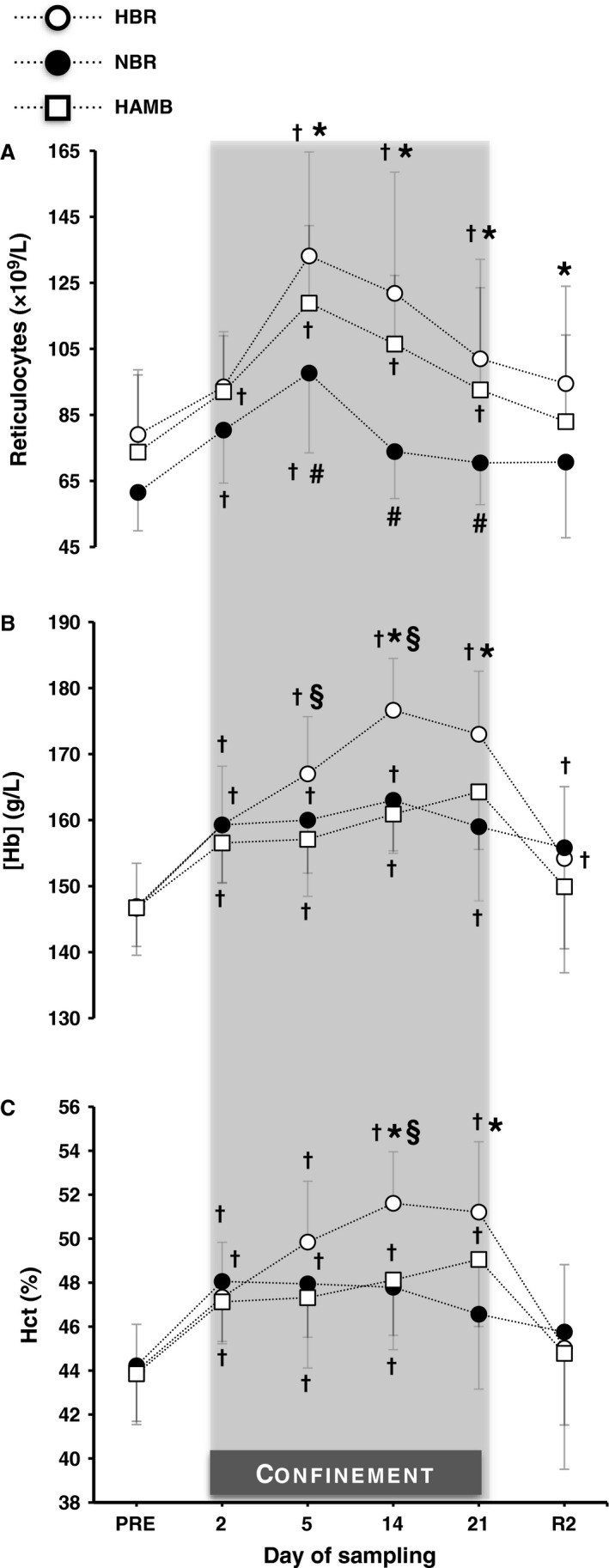
Reticulocyte counts, hemoglobin concentration ([Hb]), and hematocrit (Hct) before (Pre), during (day 2, 5, 14 and 21), and after (R2) the 21‐day hypoxic bed rest (HBR), normoxic bed rest (NBR), and hypoxic ambulation (HAMB). Values are mean ± SD. ^†^Significantly different from the Pre. *Significant differences between HBR and NBR. ^§^Significant differences between HBR and HAMB. ^#^Significant differences between NBR and HAMB. (*P *≤* *0.05).

There was a confinement × time interaction (*P *=* *0.05, *η* ^2^_p_ **=* *0.18), and a main effect for confinement (*P *<* *0.001, *η* ^2^_p_ **=* *0.97) and time (*P *=* *0.001, *η* ^2^_p_ **=* *0.49) (Fig. 3A). Both hypoxic confinements caused a significant increase in reticulocytes (HAMB: *P *≤* *0.01; HBR: *P *<* *0.001). During NBR, the reticulocytes were elevated on day 2 and 5 (*P *≤* *0.01), and thereafter returned to the baseline level. From day 5 to the end of the confinement, the count of reticulocytes was constantly lower in NBR than in both hypoxic confinements (*P *≤* *0.05). During the postconfinement period, the reticulocytes were less in NBR than in HBR (*P *=* *0.01).

### Hemoglobin

There was a confinement × time interaction (*P *=* *0.004, *η* ^2^_p_ **=* *0.34), and a main effect for confinement (*P *=* *0.002, *η* ^2^_p_ **=* *0.45) and time (*P *<* *0.001, *η* ^2^_p_ **=* *0.73) (Fig. 3B). [Hb] was significantly increased throughout the three confinements (*P *<* *0.001). However, [Hb] was greater in HBR compared to HAMB on day 5 and 14 (*P *<* *0.05), and to NBR on day 14 and 21 (*P *<* *0.001). [Hb] was still elevated on R2 after both bed rest confinements (HBR: *P *=* *0.05; NBR: *P *=* *0.01).

### Hematocrit

There was a confinement × time interaction (*P *<* *0.001, *η* ^2^_p_ **=* *0.33), and a main effect for confinement (*P *=* *0.02, *η* ^2^_p_ **=* *0.31) and time (*P *<* *0.001, *η* ^2^_p_ **=* *0.70) (Fig. 3C). Both hypoxic confinements caused an increase in Hct (*P *≤* *0.01); on day 14, the increase was greater in HBR than in HAMB (*P *=* *0.05). NBR caused an increase in Hct on day 2, 5, and 14 (*P *≤* *0.01); still, Hct was lower in NBR than in HBR on day 14 and 21 (*P *<* *0.001).

### Red blood cells

The mean values of RBCs are summarized in Table 2. There was a confinement × time interaction (*P *<* *0.001, *η* ^2^_p_ **=* *0.40), and a main effect for confinement (*P *<* *0.001, *η* ^2^_p_ **=* *0.54) and time (*P *<* *0.001, *η* ^2^_p_ **=* *0.73). RBCs were elevated throughout the three confinements (*P *<* *0.001). RBCs were higher in HBR than in HAMB on day 5, 14, and 21 (*P *<* *0.001), and than in NBR on day 14 and 21 (*P *<* *0.001).

### Plasma renin

**Figure 4:**
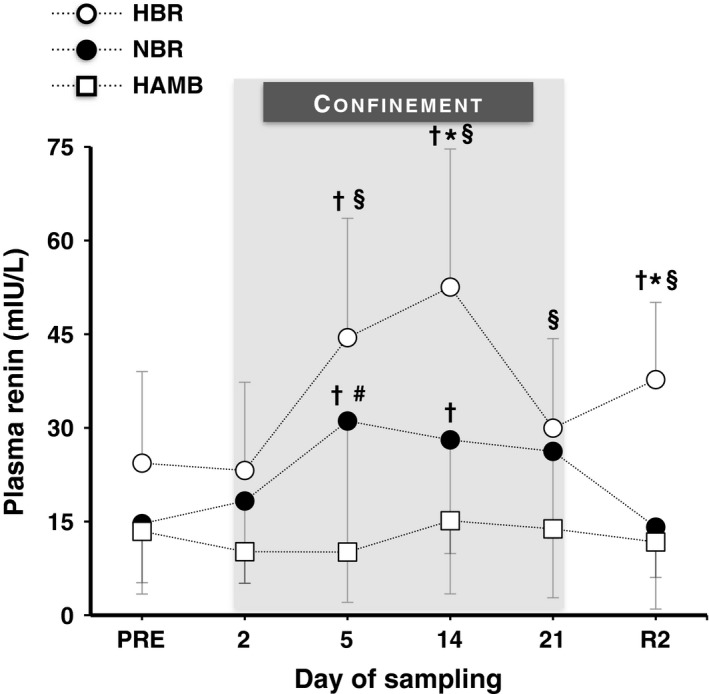
Absolute values of plasma renin concentration before (Pre), during (day 2, 5, 14 and 21), and after (R2) the 21‐day hypoxic bed rest (HBR), normoxic bed rest (NBR), and hypoxic ambulation (HAMB). Values are mean ± SD. ^†^Significantly different from the Pre. *Significant differences between HBR and NBR. ^§^Significant differences between HBR and HAMB. ^#^Significant differences between NBR and HAMB. (*P *=* *0.006).

There was a confinement × time interaction (*P *=* *0.006, *η* ^2^_p_ **=* *0.29), and a main effect for confinement (*P *<* *0.001, *η* ^2^_p_ **=* *0.78) and time (*P *<* *0.001, *η* ^2^_p_ **=* *0.42) (Fig. 4). NBR increased renin on day 5 and 14 (*P *<* *0.05). HBR enhanced renin on day 5, 14, and R2 (*P *≤* *0.001). On days 14 and R2, the increase was greater in HBR than in NBR (*P *<* *0.001). HAMB did not alter renin at any time point (*P *>* *0.05).

### Qualitative interconfinement differences in plasma volume

**Figure 5:**
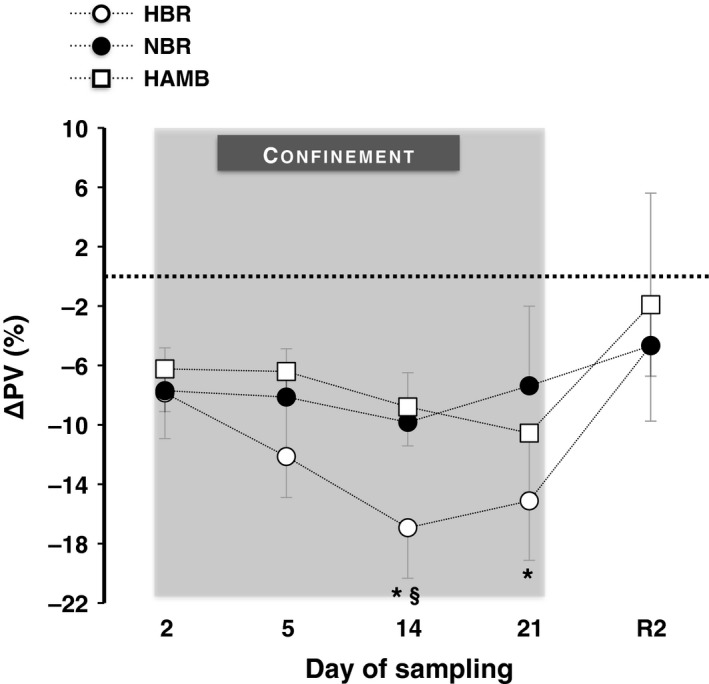
Mean changes from baseline values of plasma volume (ΔPV) before (Pre), during (day 2, 5, 14 and 21), and after (R2) the 21‐day hypoxic bed rest (HBR), normoxic bed rest (NBR), and hypoxic ambulation (HAMB). Values are mean ± SD. Data in all confinements were significantly different than the Pre. *Significant differences between HBR and NBR. ^§^Significant differences between HBR and HAMB. (*P *=* *0.008). Note that the absolute values of PV should be interpreted with caution, and that the interconfinement differences reported are also based on coinciding changes in plasma renin concentration (see Fig. 4), as described in the methods section.

The mean and individual changes from baseline values of PV are illustrated in Figure 5. There was a confinement × time interaction (*P *<* *0.008, *η* ^2^_p_ **=* *0.32), and a main effect for confinement (*P *=* *0.002, *η* ^2^_p_ **=* *0.44) and time (*P *<* *0.001, *η* ^2^_p_ **=* *0.70). All three confinements caused a significant drop in PV (*P *<* *0.05). However, the reduction in PV was greater in HBR than in NBR on day 14 and 21 (*P *<* *0.001), and than in HAMB on day 14 (*P *<* *0.001). The changes in PV were mirrored by the changes in plasma renin concentration, so that the largest drop in PV coincided with the largest increase in plasma renin and so forth (Fig. 4).

## Discussion

A substantial reduction in serum EPO concentration was observed in all subjects on the second day of NBR; a finding that is in line with a previous bed rest study (Gunga et al. 1996). Although the secretion of EPO is primarily mediated via an intrarenal O_2_‐dependent mechanism (Bauer and Kurtz 1989; Lundby et al. 2014), EPO transcription is also modulated by changes in the blood‐volume distribution in the body (Ehmke et al. 1995; Kirsch et al. 2005). During the early stages of bed rest, a thoraco‐cephalad fluid shift prevails, resulting in a rapid and transient increase in central venous pressure (Nixon et al. 1979; Linnarsson et al. 1985). It has been postulated (Gunga et al. 1996) that this central venous pressure increase is detected by the cardiac atrial volume receptors, which suppress EPO production, either by affecting the renin–angiotensin system (Freudenthaler et al. 1999, 2000; Donnelly and Miller 2001), or via a humoral modulator stemming from the hypothalamic–hypophyseal system (Pagel et al. 1989; von Wussow et al. 2005). In this regard, the report by Breymann et al. (2000) showing that the increase in central venous pressure in a group of hypervolemic hemodiluted individuals was closely associated with a reduction in EPO is also of interest. Aside from the blood volume changes, Robertson et al. (1994) have suggested that reduced sympathetic nervous activity might explain the EPO suppression during bed rest. However, judging by the arterial pressure and HR responses, NBR did not reduce sympathetic nervous activity in this study.

Notably, the hypoxic stimulus counteracted any bed rest‐induced EPO suppression, resulting in marked increase in the serum EPO during HBR. In keeping with our findings, Stevens et al. (1966) have reported that hypoxia during a 4‐week bed rest prevented a loss in red cell mass. Yet, in this study, it is noteworthy that the EPO increment in HBR was less than that detected in HAMB, despite the similar levels of P_I_O_2_ in the two confinements, suggesting that the bed rest‐induced central hypervolemia was still capable of blunting the hypoxia‐driven erythropoiesis. Thus, present results support the notions that tissue hypoxia is the prime mover of renal EPO synthesis (Jelkmann 2011), and that the blood‐volume distribution constitutes an additional regulatory determinant for EPO production.

The confinement‐dependent reduction in PV was greater in HBR than in the other conditions, which is in agreement with the suggestion by Loeppky et al. (1993). The underlying mechanisms for the exaggerated depression of PV in HBR are difficult to discern from the current results. It appears unlikely that the exaggerated PV drop was due to increased diuresis, since water intake and urine output were well preserved in all confinements and did not differ between them (see Debevec et al. 2014). Presumably, the HBR‐induced hypovolemia was attributable to a higher sympathetically mediated vascular tone, mainly induced by the hypoxic stimulus (Kanstrup et al. 1999; Hansen and Sander 2003), as indicated by the higher values of HR and DAP during HBR than NBR. An increased peripheral vasoconstriction during HBR might have prompted blood redistribution from the periphery to the central circulation, with a more pronounced raise in right atrial pressure and hence stimulating the release of ANP, and leading to a greater degree of hypovolemia. An alternative mechanism might be that HBR caused a greater loss of plasma proteins, reducing vascular osmotic pressure. Thus, both hypoxia (Sawka et al. 1996) and bed rest (Van Beaumont et al. 1972; Cirillo et al. 2002) may induce hypoproteinemia. Lastly, the increased concentration of EPO in HBR could also have contributed, at least to some extent, to the PV reduction (Lundby et al. 2007). Still, the mechanisms underlying the HBR‐induced hypovolemia remain speculative, and need to be further investigated.

Notwithstanding the EPO suppression, NBR increased reticulocytes and RBCs, responses that presumably were attributable to the bed rest‐induced PV contraction (Lawrence and Berlin 1952). Trudel et al. (2009) have also suggested that the bed rest‐induced hematopoietic stimulation might be associated with accumulation of fat in the bone marrow, leading to an increased availability of cytokines (i.e., leptin, adiponectin) locally in the hematopoietic vertebrae, which, in turn, may stimulate hematopoiesis (Bennett et al. 1996; Yokota et al. 2000). Yet, in this study, the rise of reticulocytes was apparent already 2 days after the initiation of bed rest, which seems an insufficiently short time period to induce significant fat accumulation in hematopoietic bone marrow. Of note is, however, that HBR, despite resulting in a less pronounced [EPO] elevation, caused a greater increase in reticulocytes and RBCs than did HAMB, suggesting that HBR‐induced hematopoiesis may have been of mixed origin, and not solely EPO dependent; the mechanisms need to be further elucidated.

HAMB reduced the plasma renin by ~25% on days 2 (*d *=* *0.41) and 5 (*d *=* *0.36), albeit the difference was not statistically significant (*P *=* *0.99): it was reduced in eight and increased in three subjects. Indeed, the findings regarding the effect of prolonged hypoxic exposure on renin are equivocal; a few studies have reported a reduction (Hogan et al. 1973; Humpeler et al. 1980; Siebenmann et al. 2015), whereas others have shown either a rise (Slater et al. 1969; Milledge et al. 1983a,b), or no change (Sutton et al. 1977; Keynes et al. 1982; Robach et al. 2000). Considering that the response of renin to hypoxia is dependent on the degree of physical activity (Milledge et al. 1983a,b), the interindividual variability observed in the present HAMB confinement might be explained by the exercise regimen in this condition. Conversely, HBR and NBR caused a significant increase in the renin concentration, which was most likely mediated by the level of hypovolemia (Gauer and Henry 1976); the increase was more profound in HBR due to the greater PV drop. Furthermore, after the cessation of HBR, the concentration of renin was significantly higher than in the preconfinement period. This “paradoxical” overshooting, which has also been observed following a prolonged high‐altitude exposure (Robach et al. 2000), might be ascribed to a transient imbalance of two conflicting stimuli, the inhibition of the hypoxia‐induced renin suppression during reoxygenation (Siebenmann et al. 2015), and the stimulation of renin secretion during reambulation (Brown et al. 1966; Nielsen and Moller 1968).

### Methodological considerations

In this study, the interconfinement differences in PV changes were graded qualitatively by comparing PV changes as estimated indirectly from changes in [Hb] and Hct (Dill and Costill 1974) with the concomitant changes in plasma renin concentration (see Materials and Methods). Although the Dill and Costill method (Dill and Costill 1974) might underestimate the actual changes in PV at the early phase of postural changes (Johansen et al. 1998), it accurately reflects the PV changes during a prolonged period of normoxic bed rest, provided that the horizontal, supine position is used as the reference (Johansen et al. 1997). Indeed, in this study, blood draws were performed in the morning, while subjects rested in a horizontal supine position, according to standard operating procedures. It should be taken into account, however, that any neoerythrocytosis occurring during the hypoxic confinements may have led to an overestimation of PV values derived by the Dill and Costill equation (Dill and Costill 1974) during the later phase of HAMB and HBR. It nevertheless appears clear that hypoxia aggravated the bed rest‐induced PV reduction, as a substantially more pronounced elevation in plasma renin concentration was observed during HBR than NBR. Even though it appears clear that the PV drop was larger in HBR than NBR, it must be emphasized that, for the aforementioned reasons, the methods employed do not permit us to draw firm conclusions regarding the exact magnitude of PV changes. It should hence be considered to use a direct tracer‐dilution method to study PV changes in any future hypoxic bed rest studies.

Based on the topical debate regarding potential differences between normobaric and hypobaric hypoxia (cf. Millet et al. 2012; Mounier and Brugniaux 2012), present results are pertinent only to normobaric hypoxia conditions, and it remains to be settled whether the responses reported herein would be identical in hypobaric hypoxic circumstances. Lastly, in view of the evidence that the bed rest‐induced hypovolemia might be less in women than men (Fortney et al. 1994), the fluid distribution in females during a prolonged period of hypoxic bed rest needs to be examined.

In conclusion, present findings demonstrate that the bed rest‐induced suppression of serum EPO concentration is reverted by hypoxia, whereas hypoxia seems to exaggerate the bed rest‐induced reduction in PV.

## Conflict of Interest

None declared.
